# Stress and the gut-brain axis: an inflammatory perspective

**DOI:** 10.3389/fnmol.2024.1415567

**Published:** 2024-07-18

**Authors:** Julia Morys, Andrzej Małecki, Marta Nowacka-Chmielewska

**Affiliations:** Laboratory of Molecular Biology, Institute of Physiotherapy and Health Sciences, Academy of Physical Education, Katowice, Poland

**Keywords:** gut-brain axis, blood-brain barrier, tight junctions, stress, inflammation, mood disorders

## Abstract

The gut-brain axis (GBA) plays a dominant role in maintaining homeostasis as well as contributes to mental health maintenance. The pathways that underpin the axis expand from macroscopic interactions with the nervous system, to the molecular signals that include microbial metabolites, tight junction protein expression, or cytokines released during inflammation. The dysfunctional GBA has been repeatedly linked to the occurrence of anxiety- and depressive-like behaviors development. The importance of the inflammatory aspects of the altered GBA has recently been highlighted in the literature. Here we summarize current reports on GBA signaling which involves the immune response within the intestinal and blood-brain barrier (BBB). We also emphasize the effect of stress response on altering barriers' permeability, and the therapeutic potential of microbiota restoration by probiotic administration or microbiota transplantation, based on the latest animal studies. Most research performed on various stress models showed an association between anxiety- and depressive-like behaviors, dysbiosis of gut microbiota, and disruption of intestinal permeability with simultaneous changes in BBB integrity. It could be postulated that under stress conditions impaired communication across BBB may therefore represent a significant mechanism allowing the gut microbiota to affect brain functions.

## 1 Introduction

The bidirectional communication network between the central nervous system (CNS) and the gastrointestinal tract, known as the gut-brain axis (GBA) was found to play a substantial role in the etiopathogenesis of many diseases, including neurodegenerative diseases, and mood disorders. The GBA involves the integration of gut functions (including immune activity or intestinal permeability) with the emotional and cognitive centers of the brain (Carabotti et al., 2015). The existence of complex gut-brain communication is supported by several animal and human studies, although the underlying mechanisms are not fully elucidated. Research in this field is focused on neural (Cryan et al., 2019), neuroendocrine (Kasarello et al., 2023), immune (Rutsch et al., 2020), and metabolic pathways (Ahmed et al., 2022). These consist of autonomic (parasympathetic) nervous system signaling via the vagal nerve, enteric nervous system, innate and adaptive immune responses, neurotransmitters (or false neurotransmitters known as neurotransmitter-like compounds) or short chain fatty acids (SCFAs) being also metabolites or products of microbial functioning (Cryan et al., 2019).

Recently, it was proposed that besides the critical role of the gut microbiota as a component potentially influencing all these neuroimmune-endocrine pathways, it can affect the integrity of the blood-brain barrier (BBB), by changing barrier permeability and dysregulating tight junctions (TJs) (Braniste et al., 2014; Margolis et al., 2021). Impaired communication across the BBB may therefore represent a significant mechanism allowing the gut microbiota to affect brain functions, for example under stress conditions.

The GBA remains under the continuous influence of environmental factors, from early life through the entire lifespan, also during acute or chronic stress conditions (De Palma et al., 2015; Golubeva et al., 2015; Brzozowski et al., 2016). The role of immune signaling in neuronal development has also been thoroughly studied (Kipnis et al., 2004). Moreover, it was determined that presence of immune T cells, mediated partially by gut microbiota, has a vital role in microglia maturation (Pasciuto et al., 2020). Even short-term exposure to stress can induce dysbiosis. This term refers to alterations in microbiome composition, which leads to disturbed homeostasis, metabolic, and functional changes as the consequence of shifts in bacteria species residing in the gut (Levy et al., 2017). Gut dysbiosis is associated with the progress or worsening of mood disorders caused by various aspects of GBA functioning, such as activation of immune signaling during inflammatory processes. It is now well established that CNS disorders as well as diseases directly associated with the gut have strong stress-based pathogenesis (Holmqvist et al., 2014; Gao et al., 2018). Importantly, experimental alteration of gut microbiota influences stress responsiveness, anxiety- and depressive-like behavior, and activates the hypothalamic-pituitary-adrenal (HPA) axis (De Palma et al., 2014; Golubeva et al., 2015; Ergang et al., 2021). Significant shifts in gut microbiota composition reported in animal models of CNS diseases related to early life stress, such as maternal separation (De Palma et al., 2015), chronic restraint stress (CRS) (Gubert et al., 2020) or chronic unpredictable mild stress (CUMS) (Zhang et al., 2023) were linked with alterations in microbiota-related metabolites and immune signaling pathways suggesting that these systems may be important in stress-related conditions including depression.

Therefore, the aim of this review paper is to summarize findings linking the gut microbiota and brain health with special emphasis on inflammation and immune signaling pathways. We highlight current studies on inflammatory aspects of altered GBA functioning and deliver insights on its association with neuroinflammation and mood disorder development. So far, many authors have developed the concept of GBA, which undoubtedly contributed to increasing awareness of its role in systemic homeostasis (Carabotti et al., 2015; Cryan et al., 2019; Rutsch et al., 2020; Margolis et al., 2021). However, one of the main threads that distinguishes this work is the focus on the aspect of fluctuations within the BBB. We discuss the impact of metabolites generated by gut microbes on the BBB integrity, the involvement of stress exposure in intestinal barrier leakiness, and further changes in BBB permeability. Finally, we summarize the data from animal studies employing the therapeutic potential of manipulating the gut microbiota through probiotic, synbiotic, or fecal microbiota transplantation in the context of CNS disorders.

## 2 Gut barriers and the blood-brain barrier

**Figure 1:**
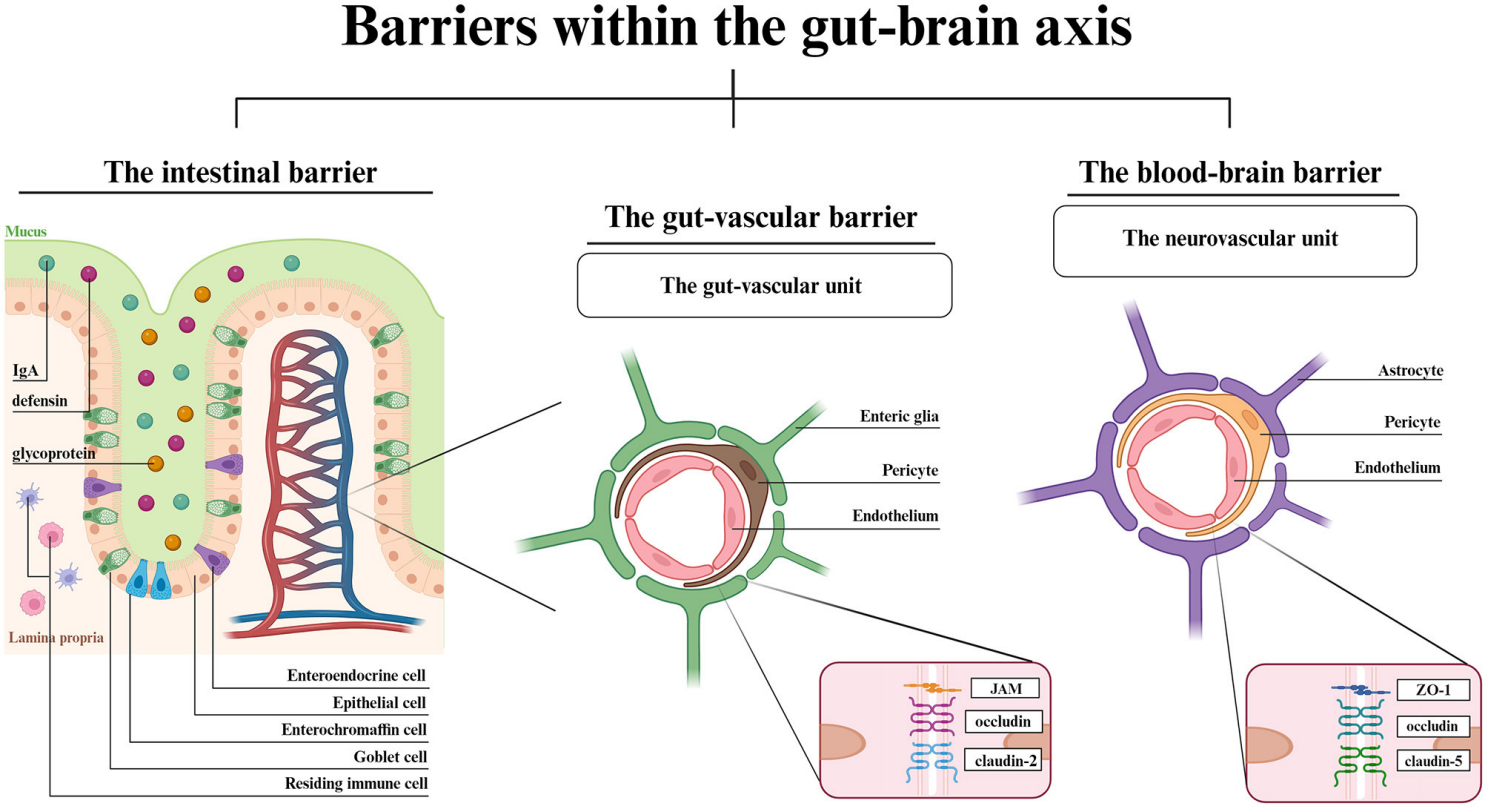
Barriers within the GBA- a structural overview. The IB is composed of epithelial cells with goblet, enterochromaffin and enteroendocrine cells in between. The IgA, defensins and glycoproteins are main components of mucosal layer. Dendritic cells and macrophages reside in the intestinal lamina propria. The vascular barriers are both composed of endothelial cells, pericytes and glial cells. Each vascular unit shows the most highly expressed junctional proteins. Created with BioRender.com.

Apart from molecular mechanisms engaged in GBA signaling, this axis also stands for physical barriers surrounding the gastrointestinal tract and CNS (Figure 1).

The lumen of gastrointestinal tract is separated from the blood circuitry by an intestinal barrier (IB) and the transport into blood vessels is controlled by the gut-vascular barrier (GVB) (Di Tommaso et al., 2023). The CNS is covered by two barriers interconnecting the blood and the brain, the BBB, and the blood-cerebrospinal fluid barrier, which involves choroid plexus epithelium (Kim et al., 2021; Hall and Bendtsen, 2023). Starting from the gut lumen surface, the IB is composed of the mucus-producing goblet cells, positioned between the epithelial cells (Suzuki, 2020). The crypt mucosal coverage, which is composed of secretory IgA, defensins and glycoproteins, and lamina propria with residing immune cells protects the gut wall from harmful pathogens (Suzuki, 2020; Kim et al., 2021). The epithelial layer has a similar function to the BBB endothelial layer. Both the epithelial and endothelial cells allow the passage of required molecules via either paracellular (IB) or transcellular (IB, BBB) active and vesicular transport. The paracellular passage in BBB is a symptom of disturbed homeostasis (Bauer et al., 2014; Haddad-Tóvolli et al., 2017). The vascular barriers, GVB and BBB are similar in structure. Both the gut-vascular unit and neurovascular unit consist of an endothelial layer wrapped in pericytes along with either enteric glial cells (GVB) or astrocytes (BBB) (Kadry et al., 2020; Di Tommaso et al., 2023). The endothelial cells are located on the luminal surface and are tightened by TJs, which modulate the barrier permeability thereby mediating signaling pathways (Bauer et al., 2014). The major difference between GVB and BBB is the eight times larger molecule flux allowed by GVB (Scalise et al., 2021). The reason for such distinction is associated with the gut functionality that involves nutrient absorption. Though the TJs and adherens junctions (AJs) expression vary among different cell types, claudins have been established to be the key components for changing barriers' permeability (Banks and Erickson, 2010; Scalise et al., 2021), claudin-5 being the most abundant throughout the BBB and in lymphatic endothelial cells of small and large intestine (Greene et al., 2019). Indeed, there are examples of expression similarity such as claudin-1, claudin-12, ZO-1, ZO-2 (TJs) or VE-cadherin and α- or β-catenin (AJs), both in the brain and in the gut (Scalise et al., 2021). The BBB anatomy enables the ionic and metabolic exchange homeostasis, proper neurotransmitter uptake and signaling mediation, and the whole barrier integrity, while the TJs and AJs between adjacent cells restrict paracellular transport and protect the brain parenchyma from unwanted hydrophilic particles passage (Scalise et al., 2021; Park and Im, 2022). In turn, the GVB adjusts its permeability to the current state of the intestinal environment and allows the passage of needed molecules while maintaining protection from adverse molecules.

## 3 Focusing on the immune system- related signaling pathways

Non-specific innate immunity is built on granulocytes, monocytes, macrophages, dendritic and mast cells as well as natural killer cells. These cells present the pattern-recognition receptor protein family, which includes Toll-like and NOD-like receptors (TLRs, NLRs), and act via inflammasome—an oligomeric protein complex. An inflammasome distinguishes pathogen/microbial/damage-associated molecular patterns present on the pathogenic cell surface, from host cell antigens (Ghilas et al., 2022). The recognition of specific pathogen-associated molecular patterns is associated with inflammasome mobilization, proinflammatory cytokines, IL-1β, and IL-18 release into the bloodstream, along with pyroptosis induction (Cryan et al., 2019; Taguchi and Mukai, 2019; Sahoo, 2020). The most studied transmembrane Toll-like protein is TLR4, which is proven to interact with bacteria-derived lipopolysaccharide (LPS) which triggers microglia—the most abundant immune cells residing in the brain. Apart from glial cells, the perivascular macrophages are the only endogenous immune cells in the CNS (Prinz and Priller, 2017). The microbiota-microglia reaction might be the cause of neuroinflammation and BBB disruption (Banks and Erickson, 2010). On the other hand, in homeostatic condition highly specific adaptive immune response evolves throughout the host's lifespan and includes T and B lymphocytes. In a recent study, the CD4^+^ and CD8^+^ T lymphocytes were observed in the brain and accessed brain parenchyma, leading to BBB altered permeability, and neuroinflammation (Park and Im, 2022).

Some unresolved questions remain as to the mechanism(s) of communication between the gut microbiota and the brain, but three major pathways have been established as involved in the signal transmission within the immune system in the GBA: the systemic, cellular, and neuronal pathways.

**The systemic pathway**, also known as the humoral pathway, focuses on the effect of local alterations either in the brain or in the gut, on the overall state of homeostasis. The altered IB and GVB permeability results in the inflammatory factors flux, bacterial translocation, and other triggers that cause the immune response, into the bloodstream (Kinashi and Hase, 2021; Mou et al., 2022). Persistent systemic chronic inflammation might affect the BBB structure, leading to its disruption, which facilitates access to the brain parenchyma (Rochfort et al., 2014). The glial cells react by pro-inflammatory (IL-1β, IL-18, IL-6, TNF-α) or anti-inflammatory (IL-10, IL-4, TGF-β) cytokines release, depending on the current state of the immune response (Nagyoszi et al., 2015). Recent animal studies showed elevated levels of TNF-α in rats (Chen et al., 2019), and IL-1β in nestin-Cre mice (Wong et al., 2019) in inflammation-induced BBB disruption models. IL-6 and IL-18 were found to promote hippocampal stem cell apoptosis and induce further inflammation in NZB/W-F1 lupus-prone mice (Nikolopoulos et al., 2023). A recent study performed on CD-1 mice confirmed the augmentation of CCL2 and CCL5 chemokines during inflammation and pointed out that it is the CCL2 and CCL5 ligands might have the ability to cross an intact BBB (Quaranta et al., 2023). In a mice model of intracerebral hemorrhage, Lin et al. (2023) showed that LPS ip. injection enhanced the activity of the CCL5 signaling pathway which was associated with increased permeability of the BBB.

The systemic pathway is linked with the HPA axis. As a result of peripheral (intestinal) inflammation, the HPA axis might be activated, resulting in the release of glucocorticoids (GCs) (Misiak et al., 2020). The impact of GCs overproduction on the alterations in microbiota composition was proven in animal studies (Petrosus et al., 2018; Couch et al., 2023). It is suggested that because of HPA axis-induced microbiota perturbations, the inflammatory response is activated via Th17 lymphocytes, which are also a part of the cellular pathway (Sun C. Y. et al., 2023).

**The cellular pathway** is focused on intestinal immune cells function. In an afferent way, intestinal immune cells can migrate into the CNS to promote or inhibit neuroinflammation. The efferent signaling might start from the stress-induced microbiota alteration, leading to immune response activation, immune cells mobilization and translocation to the brain parenchyma, worsening the already existent neuroinflammation (Rochfort et al., 2014). The cells engaged in intestinal immune response are myeloid cells, such as macrophages and dendritic cells, residing in lamina propria, responsible for promoting proinflammatory cytokines release (Ghilas et al., 2022). Myeloid cells also mediate Th17 responses employing the Th17 lymphocytes, a subset of CD4+ T cells. Moreover, Foxp3+RORγt+ regulatory T (Treg) cells of intestinal origin, were found to express similar gene patterns to Th17 cells, and so, were extensively studied regarding their properties. The either proinflammatory or suppressive phenotype of these cells, was proven to be dependent on the cytokine milieu, TGF-βand the type of myeloid cells that partake in T cell priming (Zhou et al., 2008; Sun C. Y. et al., 2023). In a study by Yang et al. (2016) the suppressive phenotype of Foxp3+RORγt+ Treg cells in a colitis mouse model was confirmed. -Pro-inflammatory cytokines overexpression, especially IL-1, IL-6, and IL-23 was determined to participate in the initiation of autoimmune processes (Yasuda et al., 2019). In a healthy host, gut-derived interferon γ positive natural killer (NK) cells (IFNγ+ NK) were proven to promote TRAIL+ and LAMP1+, anti-inflammatory astrocytes, responsible for neuroimmune regulation via T cell apoptosis (Sanmarco et al., 2021). Additionally, naïve B cells differentiate into IgA-producing plasma cells after an antigen encounter (Rojas et al., 2019; Fitzpatrick et al., 2020; Keppler et al., 2021). The migration pattern of these cells has not been specified yet, nonetheless, they have been repeatedly found in the BBB, playing a vital role in preventing pathogens from entering the brain parenchyma (Rojas et al., 2019; Jacobson et al., 2021). In a recent study, Brioschi et al. (2021) determined the presence of B cells in diverse development stages in mouse meninges, through which the B cells might be recruited. The lymphoid B cells were also closely linked with enteric glia and innate immunity, mediated by neurotrophic factors (Ibiza et al., 2016).

**The neuronal pathway** is associated with the autonomic nervous system and the enteric nervous system (ENS). In homeostasis enteric glial cells interact with intrinsic sensory neurons, interneurons and motor neurons, and satellite glia, located in the dorsal root ganglia (Morales-Soto and Gulbransen, 2019; Fung and Vanden Berghe, 2020; Dowling et al., 2022). During inflammation, increased S100β protein expression in enteric glia was proven to upregulate iNOS expression in T cells, macrophages, and dendritic cells (Esposito et al., 2007). Enterochromaffin cells are responsible for 5-HT (5-hydroxytryptamine) production (Reigstad et al., 2015; Rao and Gershon, 2016; Malinova et al., 2018). Enteroendocrine cells modulate signaling between enteric glial cells and GVB (Dowling et al., 2022), release cholecystokinin or YY peptide (Hayashi et al., 2023), and partake in microbial metabolites conversion such as tryptophan, and further, kynurenine (Ye et al., 2021; Zhao et al., 2023). The ENS detects bacteria-derived LPS via highly abundant TLRs, especially TLR2 and TLR4 (Hyland and Cryan, 2016).

## 4 Short-chain fatty acids (SCFAs) in the GBA

Emerging evidence suggests that gut dysbiosis and microbiota-derived metabolites significantly impact the GBA (Dalile et al., 2019). Currently, most of the research is conducted on SCFAs: acetate, propionate, and butyrate. In microbiota-depleted mice, the enteric neurons and glia loss were observed, which was then restored by SCFAs (Vicentini et al., 2021). SCFAs were also determined to mediate microglia maturation in germ-free (GF) mice (Erny et al., 2015, 2021).

SCFAs are products of fiber fermentation by gut microbiota (Martin-Gallausiaux et al., 2021; Tan et al., 2023). Therefore, in a fiber deficiency mouse model, altered cognition, hippocampal synaptic loss, and impaired brain aging were established (Shi et al., 2021). A study on butyrate and a high-fiber diet confirmed the amelioration of aging-associated neuroinflammation in mice (Matt et al., 2018). Dietary enrichment with fiber resulted in restrained inflammation (Caetano-Silva et al., 2023). In aging mice, whose microglia shifted to pro-inflammatory phenotype, dietary fiber was found to reverse this effect by increasing the SCFAs levels, thereby lowering the inflammatory features (Vailati-Riboni et al., 2022). This phenomenon was also confirmed in a microglial cell culture (Wenzel et al., 2020). An acetate shortage in type 1 diabetes (T1D) mouse model was proven to enhance cognitive impairment and aggravate hippocampal synaptophysin (SYP) expression, associated with synaptic plasticity (Zheng et al., 2021). Bacteria-derived propionate was confirmed to improve neuroregeneration (Serger et al., 2022). Based on the poststroke recovery study conducted on the murine model, SFCAs functioned as the direct mediators of T-cell functioning and microglia activation (Sadler et al., 2020).

## 5 The role of BBB and neuroinflammation in GBA dysregulation

The impairment of the BBB is one of the features associated with neuroinflammation in addition to the widely described activation of glial cells (microglia and astrocytes). The role of microglial activation in the neuroinflammation process and its interaction with BBB was studied in a mouse model of chronic systemic inflammation (MRL/lpr mice). Using *in vivo* imaging, the authors showed that microglial activation starts with glial migration to the vessel to protect the intact BBB. As the inflammation progresses, the expression of phagocytic markers (AIF-1, CD68) increases, leading to a microglial phenotype shift. Then, the AIF-1+ CD68+ microglial cells damage the BBB components, and the permeability increases which causes widespread neuroinflammation (Haruwaka et al., 2019). Chronic intestinal inflammation was found to induce dysbiosis in the gut and subsequently become systemic over time (Thevaranjan et al., 2017). As in this chronic inflammation state, a persistent microglia mobilization was observed, along with the immune cells flux which resulted in neurotoxicity and damage to the brain (Cowan and Petri, 2018; Guo et al., 2022; Brandl and Reindl, 2023). Thus, microglia-induced neural cytotoxicity, of gut microbiota origin as well, can lead to neurodegenerative and mood disorders development (Megur et al., 2020; Xia et al., 2023).

Regarding the association between neuroinflammation and altered BBB integrity, several studies focused on the potential role of SCFAs in BBB permeability. In a study by Li et al. (2023), mice with an induced sepsis-associated encephalopathy were treated with SCFA supplementation. It was confirmed that the SCFAs caused an increased expression of ZO-1 and occludin expression in the intestine and BBB. In research on GF (germ free) and SPF (specific pathogen-free) mice, authors observed the lower expression of occludin and claudin-5 in the frontal cortex, striatum, and hippocampus in both sexes of GF mice, suggesting an association between lack of microbiota and increased BBB permeability (Braniste et al., 2014). In LPS-exposed cerebromicrovascular endothelial cell culture, ZO-1 and occludin expression increased after SCFA treatment in comparison with the LPS-exposed but not treated one (Li et al., 2023). It was also determined that restoration of gut microbiota and microbial metabolites, such as SCFAs, alters TJs expression and BBB permeability (Braniste et al., 2014). The modulatory effects of SCFAs on BBB integrity were confirmed in the *in vitro* study employing a human endothelium cell line (hCMEC/D3), showing that propionate protected the BBB from inflammation by inhibition of TLR-specific pathways as well as from oxidative stress via NRF2 signaling (Hoyles et al., 2018).

## 6 Dysregulation of the GBA in stress

Chronic stress is widely acknowledged as a predisposing or precipitating factor in neuropsychiatric diseases (Becker et al., 2007). There is a clear relationship between disturbances induced by stressful stimuli, especially long-lasting, and cognitive deficits in rodent models of affective disorders (Knapman et al., 2010). Chronic stress activates the HPA axis and sympathetic nervous system, stimulating the release of catecholamines and GCs (Misiak et al., 2020) that alter the integrity of BBB. Changes in BBB integrity were associated with the promotion of depression-like behaviors in male mice (Dion-Albert et al., 2022), indicating a link between neurovascular pathology and stress vulnerability. Stress-susceptible mice following chronic social defeat stress showed depressive-like behaviors which were associated with altered BBB integrity through a decline in claudin-5 expression in the nucleus accumbens (Menard et al., 2017; Dudek et al., 2020). Additionally, the loss of tight junction proteins promoted increased peripheral IL-6 passage across BBB and monocyte accumulation which participated in the development of depression (Menard et al., 2017). Concomitant increases in intestinal permeability may intensify these effects (Braniste et al., 2014). Stress-induced impairments of intestinal barrier function (e.g., changes in TJs protein expression) contribute to increased intestinal permeability and the movement of antigens and factors, such as LPS, from the gut lumen into the circulation, which exacerbate the immune response (Dinan and Cryan, 2012; Yang et al., 2023). Recently, chronic stress inducing a downregulation of intestinal and hippocampal expression of α-actin, claudin-1, claudin-5, occludin, and ZO-1 was observed along with altered microbial diversity in the gut (Geng et al., 2020; Chi et al., 2021). Compromised IB integrity was accompanied by seral declined levels of 5-HT, GABA, and increased levels of endotoxin, IL-6, and TNF-α as well as increased IL-6, NF-κB, iNOS, and NGAL in the intestine (Chi et al., 2021). In turn, disrupted BBB correlated with increased norepinephrine levels in the prefrontal cortex (PFC), hippocampus, and amygdala of female SPF mice (Geng et al., 2020). Consequences of peripheral inflammation, such as disruption of BBB integrity (de Vries et al., 1996) and stimulation of neuroinflammation (Geng et al., 2018), have been associated with cognitive impairments and mood disturbances.

### 6.1 Chronic unpredictable mild stress

The role of stress exposure in GBA dysregulation is widely explored in the literature. However, the influence of stress response on intestinal barrier leakiness, and further changes in BBB permeability were investigated only in a few experimental studies (summarized in Table 1). Most research performed in various stress models shows that functional changes (anxiety- and depressive-like behaviors) correlated with dysbiosis of the GBA, and IB disruption with an enhanced peripheral inflammatory response was associated with changes in BBB permeability and neuroinflammation. The impact of stress on GBA functions in the context of BBB changes is most commonly described in chronic stress paradigms (Liang et al., 2015; Bharwani et al., 2017; Nie et al., 2019; Yang et al., 2021; Jiang et al., 2023; Kitaoka et al., 2023), especially by employing social stressors (chronic social stress, or CUMS). CUMS-induced behavioral impairments were associated with the downregulation of intestinal TJs, upregulation of intestinal inflammatory factors followed by altered microbial diversity, and an increase in cortical and hippocampal microglia activation (Ait-Belgnaoui et al., 2014; He et al., 2024).

**Table 1:** Summarized results of research on stress response influence on IB and BBB permeability.

**Stress model**	**Animals**	**Brain region**	**Outcomes**	**Gut**	**Outcomes**	**Gut dysbiosis**	**Behavioral impairments**	**Reference**
Chronic restraint stress	Male (M) and female (F) C57BL/6J mice (6-8 weeks old)	Hippocampus	↓ occludin, claudin-2, claudin-8 ↑ ZO-1 expression (F,M) ↑ IL-1β, IL-6 (F) ↑ IL-1β (M)	Proximal colon Distal colon	↓ occludin, claudin-2, claudin-8 (F) ↑ ZO-1 expression (F,M) ↑ IL-1β, IL-6 (F)	YES	Anxiety-like behaviors depressive-like behaviors	Jiang et al., 2023
	Male C57Bl/6N mice (6 weeks old)	PFC hippocampus	Kynureine pathway disruption alterations in Trp pathway and 5-HT levels	Duodenum Jejunum Ileum Colon Cecum	Kynureine pathway disruption alterations in Trp pathway and 5-HT levels altered intestinal crypt integrity ↓ ZO-1 expression in the ileum and colon ↑ inflammatory cell infiltration of the small intestine and goblet cell damage	YES	Anxiety-like behaviors depressive-like behaviors	Deng et al., 2021
Chronic mild unpredictable stress	Male C57Bl/6N mice (6-8 weeks old)	Forebrain hypothalamus amygdala hippocampus	↓ expression of neurotrophic protein coding genes ↑ microglia- related gene expression	Colon	↑ intestinal permeability (Cr-EDTA passage) ↓ occludin and JAM-A expression	Not measured	Not measured	Ait-Belgnaoui et al., 2014
	Male C57Bl/6N mice (8 weeks old)	PFC amygdala hippocampus	↑ microglia activation in amygdala and hippocampus ↑ expression of immune-related genes in PFC (*Ccl2, Ccl5, Tlr4, Icam, Vcam, Casp1*)	Ileum	↑ ileal kynureine levels ↑ ileal levels of IL-17A, IL-1β and IL-6 ↑ ileal Th17 and Treg levels	YES	Anxiety-like behaviors depressive-like behaviors	Westfall et al., 2021b
	Male C57Bl/6N SPF mice (8 weeks old)	PFC hippocampus	↑ levels of hippocampal microglial activation ↑ activated microglia in PFC and hippocampus ↓ hippocampal synaptic plasticity	Colon	↓ claudin-2/4, occludin and ZO-1 ↑ genes associated with immune response ↑ IL-1β, TNF-α, TLR4 and 5, NF-κB, NOD-like receptor family, NLRP3, indoleamine-2, IDO-1	YES	Depressive-like behaviors	He et al., 2024
	Male Sprague-Dawley (SD) rats (8 weeks old)	PFC hippocampus	↓ occludin and ZO-1 expression ↑ ASC, caspase-1, NLRP3 expression	Colon	↓ occludin and ZO-1 expression ↑ ASC, caspase-1, NLRP3 expression	YES	Not measured	Huang et al., 2023
Chronic social stress	Male C57BL/6J mice (8 weeks old)	PFC hippocampus	↑ IL-1β in hippocampus ↑ NLRP3 expression	Ileum	↑ IL-1β ↑ TLR4 expression	Not measured	Anxiety-like behaviors depressive-like behaviors	Westfall et al., 2021a
Chronic psychological stress	Female SPF mice (4 weeks old)	PFC amygdala hippocampus	↓α-actin, claudin-5, occludin, ZO-1 expression in amygdala and hippocampus ↑ norepinephrine levels	Duodenum Jejunum Ileum	↓α-actin, claudin-5, occludin, ZO-1 expression	YES	Not measured	Geng et al., 2020
Chronic noise exposure	Male APP/PS1 and C57BL/6Nj mice (8 weeks old)	Hippocampus	↓ expression of claudin-1, occludin and ZO-1 ↑ levels of Aβ40 and Aβ42 ↑ tau phosphorylation	Colon	↓ expression of claudin-1, occludin and ZO-1 ↑ IL-6, NF-κB, iNOS, and NGAL	YES	Not measured	Sun et al., 2019
Single prolonged stress	Male (M) and female (F) Sprague–Dawley rats (6-7 weeks old)	PFC hippocampus	↓ claudin-5 expression	Cecum	↓ acetate level (M)	YES	Anxiety-like behavior	Tanelian et al., 2024
Acute psychological stress	Male (M) and female (F) Nod1/Nod2 double knockout (NodDKO) mice (6-8 weeks old)	PFC hippocampus	↓ neural activation and cell proliferation in NodDKO mice hippocampus ↓ 5-HT signaling in hippocampus ↑*Gabab1b* expression in PFC	Ileum Colon	↑ intestinal barrier permeability in NodDKO mice [FITC flux]	Not measured	Social deficits anxiety-like behavior	Pusceddu et al., 2019
Acute sleep deprivation	C57BL/6 J mice (7 weeks old)	PFC cerebral cortex hippocampus	↑ TNF-α expression in PFC ↓ 5-HT receptor expression in hippocampus ↑ TNF-α, ICAM1, IL-6 expression in cerebral cortex	Proximal colon	↓ occludin expression ↑ intestinal permeability [LPS migration] ↑ TNF-α expression	YES	Anxiety-like behavior	Yang et al., 2023
Early life stress	Male (M) and female (F) C3H/HeNRj mice (10 weeks old)	PFC	Affected PFC gene expression of over 100 genes, including *Arc, Btg2, Dusp1, Egr4, Fosb, Gadd45b, H2-k1, Junb, Klf2, Nr4a3*	Colon	↑ intestinal permeability (M)	YES	Social deficits (M) anxiety- and compulsive- like behaviors (F)	Rincel et al., 2019
High temperature stress	Male C57BL/6J mice (7-8 weeks old)	PFC hippocampus	↑ NLRP3, ASC and caspase-1 expression	Cecum	↓ butyrate level	YES	Not measured	Yi et al., 2021

Additionally, Westfall et al. (2021a) showing activation of microglia and elevated cortical expression of chemokines and adhesion molecules (ICAM, VCAM) with simultaneous changes in ileal immune regulation, confirmed that stress-related behavioral impairments may depend in such way on GBA dysregulation. In the study by Rincel et al. (2019) stressed mice (multifactorial early-life adversity consisting of prenatal LPS injections, chronic maternal separation, and exposure to CUMS during lactation) showed gut dysbiosis in both sexes, with differences in behavioral impairments. In males, authors reported social deficits, while in females—increased anxiety- and compulsive-like behaviors. Sex-specific variations were also observed in response to single prolonged stress (PTSD, post-traumatic stress disorder model) in male and female rats. These differences extend beyond behavioral and physiological changes to the gut microbiota and its metabolites (Tanelian et al., 2024). The results confirm the importance of gender in the behavioral response to chronic stress and suggest that it should be considered in experimental studies regarding stress and the GBA. The identification of sex-specific alterations in gut microbiota composition, functionality, and metabolites might be important for the development of sex-specific therapeutic interventions for those vulnerable to stress-induced psychopathologies.

### 6.2 Chronic restraint stress

In the CRS model, which is widely used to recapitulate depression phenotypes in rodents, the presence of anxiety- and depressive-like behavior was connected either with the central or peripheral inflammation, including downregulation of TJs, enhanced expression of proinflammatory factors, and increased IB permeability (Deng et al., 2021; Yang et al., 2021; Jiang et al., 2023). In the study by Yang et al. (2021) CRS mice manifested elevated hippocampal levels of proinflammatory cytokines with a simultaneous decline of BDNF (brain-derived neurotrophic factor) and 5-HT, dopamine, noradrenaline, their corresponding metabolites levels, as well as gut microbiota dysbiosis, which was linked with depressive-like behavior. Notably, depressive behaviors, the altered neurotransmitter metabolism, and microbiota dysbiosis observed in CRS mice can be restored by dexamethasone administration, underscoring the key role of inflammation in gut dysbiosis in stress-induced depressive behaviors (Yang et al., 2021). In a study by Deng et al. (2021) exposure to CRS affected kynurenine pathway components in the intestinal tract and the brain structures. Downregulated ZO-1 expression caused IB disruption which resulted in enhanced inflammatory cell infiltration of intestinal crypts. Following CRS, the elevation of IL-6 levels in the proximal and distal colon, and the increase of IL-1β only in the distal section were observed in stressed females. Greater sensitivity to stress observed in females was associated with anxiety-like behavior which correlated with the abundance of specific gut microbes, increased protein levels of IL-1β, IL-6, and gene expression of ZO-1 in the hippocampus, probably because of peripheral inflammation (Jiang et al., 2023).

### 6.3 Stress-induced inflammasome activation

Signaling pathways of NLRP3 (nucleotide oligomerization domain-like receptor family, pyrin domain containing 3) inflammasome might be involved in GBA regulation in stress conditions since its inhibition has been associated with decreased BBB permeability (Bellut et al., 2021), ameliorated cognitive impairment (Zhu et al., 2021), or reprogramming of microglial phagocytic phenotype (Jia et al., 2022). Yi et al. (2021) observed a decrease in cecal butyrate, and an increase in IL-1β, IL-6, and serum TNF-α levels and activation or inhibition of central NLRP3 inflammasomes because of exposure to high- or low-temperature stress, respectively. CUMS-exposed rats showed enhanced NLRP3 activity, along with a decrease in ZO-1 and occludin expression in the colon as well as in the prefrontal cortex and hippocampus (Huang et al., 2023). Additionally, it was proposed that intestinal epithelial NLRs might be the novel modulators of the GBA. NOD1/NOD2 knockout (KO) mice, following exposure to acute psychological stress linked with increased permeability in the ileum and colon, showed increased susceptibility to HPA axis hyperactivation, cognitive impairments, anxiety- and depressive-like behaviors. These results were associated with the altered 5-HT signaling in the hippocampus, suggesting a link between 5-HT and NLR (Pusceddu et al., 2019). Following chronic cold stress, a decrease in dopamine signaling pathway-related metabolites levels was associated with increased intestinal barrier permeability and increased release of immune response mediators. Moreover, stressed mice also manifested depressive-like behaviors (Sun C. Y. et al., 2023; Sun L. et al., 2023).

### 6.4 Prebiotic, probiotic, and synbiotic administration in stress-induced dysbiosis

As the intestinal microbiota is highly susceptible to environmental fluctuations, dysbiosis restoration treatments were proposed as a strategy to alleviate behavioral impairments. Such treatments may modify gut microbiota and levels of its metabolites thus mediating the therapeutic action in stress-induced brain dysfunctions. The beneficial impacts of the oral prebiotic (Jiang et al., 2023), probiotic (Burokas et al., 2017; De Santa et al., 2024), and/or synbiotic (Westfall et al., 2021a,b) applications on stress-induced alterations were confirmed in a few experimental studies. The application of specific prebiotic types might moderate inflammation in the hippocampus and colon, and thereby improve stress-induced depressive- and anxiety-like behaviors (Jiang et al., 2023). Moreover, the treatment with probiotics was found to downregulate stress-induced CORT (corticosterone) release and proinflammatory cytokine levels, increase acetate and propionate amounts in the intestine, and significantly alleviate depressive- and anxiety-like behaviors in mice (Burokas et al., 2017). In a study by Westfall et al. (2021a,b) mice were subjected to synbiotic treatment (*L. plantarum, B. longum*) after chronic unpredictable stress exposure. The synbiotic application weakened neuroinflammation in the hippocampus and prefrontal cortex and alleviated peripheral inflammation via downregulation of proinflammatory cytokines, TLR4, and NLRP3 expression. It also decreased the Th17/Treg ratio and subsequently the IL-17/IL-10 ratio in serum, and improved depressive- and anxiety-like behaviors. In a study by De Santa et al. (2024), the multi-strain probiotic treatment reversed depressive-like, and anxiety-like behaviors induced by the maternal separation, and normalized neuroinflammation by restoring gut microbiota. Additionally, the probiotic treatment also significantly affected the production of SCFA and the level of butyrate.

### 6.5 Fecal microbiota transplant in stress- induced dysbiosis

Microbiota-induced inflammation was proven to be completely reversed by microbial restoration via fecal microbiota transplant (FMT). The goal of FMT involves direct transfer of fecal microbiota from a healthy donor to the GI of a recipient to treat the disease by restoring the phylogenetic diversity and microbiota of a healthy person. In a few animal models of the Alzheimer's disease, FMT was shown to be effective not only in restoring gut microbiota but also in ameliorating cognitive impairments, and downregulating Aβ plaques accumulation (Sun et al., 2019; Kim et al., 2020; Elangovan et al., 2022; Hang et al., 2022). The FMT effectiveness was also determined in Parkinson's disease animal models via alleviating LPS-induced neuroinflammation (Sun et al., 2018; Zhao et al., 2021). Currently, there is a growing emphasis on the studies that use FMT approaches to study the role of stress-related microbiota composition in behavioral changes. A link was also recently demonstrated between disease-related microbiota and behavior where FMT from depressed patients to microbiota-depleted rodents increased anhedonia and anxiety-like behaviors (Kelly et al., 2016; Liu et al., 2020). Some studies employed FMT from control and stressed animals (donors) (Marcondes Ávila et al., 2020; Sharma et al., 2020; Rao et al., 2021; Huang et al., 2023; He et al., 2024) confirming the vital role of FMT in regulating brain inflammation, and behavioral changes in the chronic stress paradigms. He et al. (2024) employed FMT from stress-resilient and stress-susceptible mice to CUMS-exposed ones. Besides the significant behavioral improvement, the FMT from stress-resilient mice regulated CX3CL1 and CD200 mediators, contributing to efficient communication between microglia and neurons. In a study by Li et al. (2019), the authors determined worsened behavioral deficits because of an FMT from CUMS-treated donors. Additionally, recipient mice exhibited increased IDO-1, TNF-α, and IFN-γ levels in the hippocampus. Marcondes Ávila et al. (2020) confirmed that chronic mild stress (CMS) aggravated behavioral impairments like the effects of FMT from stressed donors to non-stressed recipients. FMT from stress-exposed rats contributed to elevated IL-6 and TNF-α hippocampal expression. On the other hand, the downregulation of proinflammatory cytokines and behavioral development was established as a result of FMT treatment from healthy donors.

## 7 Summary

**Figure 2:**
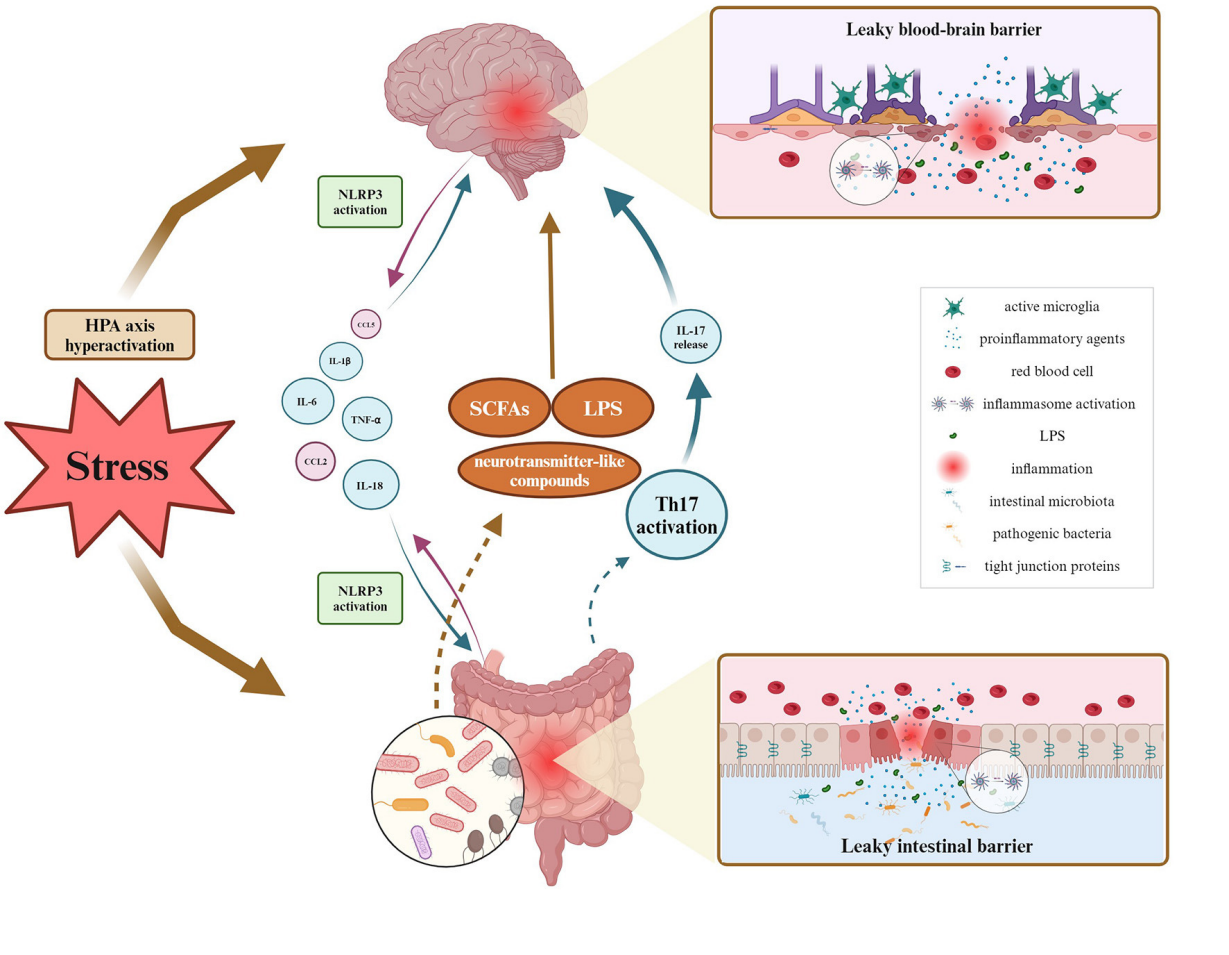
Overview of GBA fluctuations during inflammatory processes under stress stimuli. As for bidirectional communication, it has been proven so far that the leaky intestinal barrier affects the blood-brain barrier's permeability via multiple proinflammatory agents migrating within the blood circuitry, and contrariwise. Inflammation of either intestinal or brain origin, results in the NLRP3 inflammasome activation, and decreased expression of tight junction proteins and related barrier's disruption. Peripheral inflammation may activate phagocytic microglia phenotype, causing neuroinflammation. Then, neuroinflammation alters microbial diversity, thereby influencing the production and release of SCFAs, LPS, and other microbial metabolites. Created with BioRender.com.

Several studies have been exploring neuroimmune pathways involved in the regulation of the GBA in animal models of diseases, but bidirectional communication remains not fully explained. Most of the research performed in various disease models shows the involvement of the activation of the NLRP3 inflammasome complex. A few studies report the intestinal barrier's altered permeability manifested by the changes in TJs protein expression and further increased levels of cytokines and chemokines. Since behavioral impairments resulting from stress exposure are accompanied by exacerbated inflammation, special attention should be drawn to the BBB permeability. A couple of studies pointed out that stress-induced anxiety- and depressive-like behaviors correlated with dysbiosis of gut microbiota, and disruption of intestinal permeability with simultaneous changes observed in the hippocampus and prefrontal cortex. Namely, authors observed involvement of neuroinflammation processes (microglial activation, increased expression of NLRP3 and pro-inflammatory cytokines), alterations in levels of neurotransmitters, and decreased expression of ZO-1, occludin, and claudins. It could be postulated that impaired communication across BBB may therefore represent a significant mechanism allowing the gut microbiota to affect brain functions for example under stress conditions (Figure 2).

Understanding the molecular mechanisms underlying stress susceptibility seems to be crucial to the identification of novel pharmacological treatments for gut-brain disorders. Currently, scientists acknowledge the significance of changes in gut microbiota composition in mood disorders. However, using animal models to evaluate the modifications of gut microbiota (probiotics, prebiotics, FMT) requires paying special attention to the experimental design. Despite the growing use of FMT in clinical and animal studies, accompanied by scientifically proven efficiency showing anti-inflammatory properties and changes in behavior, alterations in the BBB have not been addressed so far. It remains an open question whether animal models should be used as adequate research tools to study modifications of gut microbiota under stress conditions. Therefore, drawing direct conclusions about the role of BBB integrity in the beneficial role of modification of gut microbiota is not fully possible, and it should be considered in future studies.

## Author contributions

JM: Investigation, Visualization, Writing – original draft. AM: Supervision, Writing – review & editing. MN-C: Conceptualization, Project administration, Supervision, Writing – original draft, Writing – review & editing.
